# Element partitioning of minerals and trace metals in value-added product from insect-mediated valorisation of seafood waste

**DOI:** 10.1039/d6ra06918e

**Published:** 2026-09-16

**Authors:** Shristi Upadhyaya, Michael Hayes, Victoria Vail, Damian Tweedy, Erik Neff, Jeffrey Plumlee, Damon Abdi

**Affiliations:** a School of Plant, Environmental and Soil Sciences, Louisiana State University Agricultural Center 219 Sturgis Hall Baton Rouge LA USA mhayes@agcenter.lsu.edu; b Louisiana Sea Grant, Louisiana State University Baton Rouge LA USA; c Fluker's Cricket Farm, Inc. 1333 Plantation Avenue Port Allen LA 70767 USA; d School of Renewable Natural Resources, Louisiana State University Agricultural Center 334 Renewable Natural Resources Building Baton Rouge LA USA; e Louisiana State University Agricultural Center Hammond Research Station 21549 Old Covington Hwy Hammond LA 70403 USA

## Abstract

Seafood processing generates substantial volumes of shell byproducts that are often underutilized or landfilled, resulting in massive economic and environmental costs. In many coastal regions, limited waste management infrastructure forces processors to rely heavily on landfill disposal, increasing transportation burdens and greenhouse gas emissions. However, processing byproducts such as shell residues is a mineral-rich resource, representing an underexploited opportunity for nutrient recovery and circular reuse. In this study, we evaluated the minerals and trace metal partitioning during the bioconversion of shrimp and crab shell residues using Black Soldier Fly (BSF, *Hermetia illucens*) larvae. Shell wastes were incorporated into commercial control insect feed at 25–60% inclusion levels and reared for eight days (*n* = 3 per treatment). Elemental concentrations in feed, larvae, and frass were quantified using ICP-OES to determine the enrichment patterns. The results showed calcium exhibited a substrate-driven response, with strong enrichment in frass (up to 12.15%) and moderate accumulation in larvae. Manganese and zinc showed selective larval uptake at lower inclusion levels, while higher shell inclusion shifted these elements toward frass enrichment. Aluminium, iron, boron, and barium were largely excluded from larval biomass and concentrated in frass. Across both shrimp- and crab-based treatments, frass consistently showed a higher enrichment ratio than larvae for most elements. These findings suggest that BSF bioconversion produces safe, mineral-low larval protein while simultaneously generating mineral-enriched frass for potential use as a slowly mobilized organic soil amendment.

## Abstract 2

Research using a valorisation loop in which seafood processors' waste can be utilized for feedstock and fertilizer through insect bioconversion can provide practical solutions for waste reduction and agricultural sustainability.

## Graphical abstract

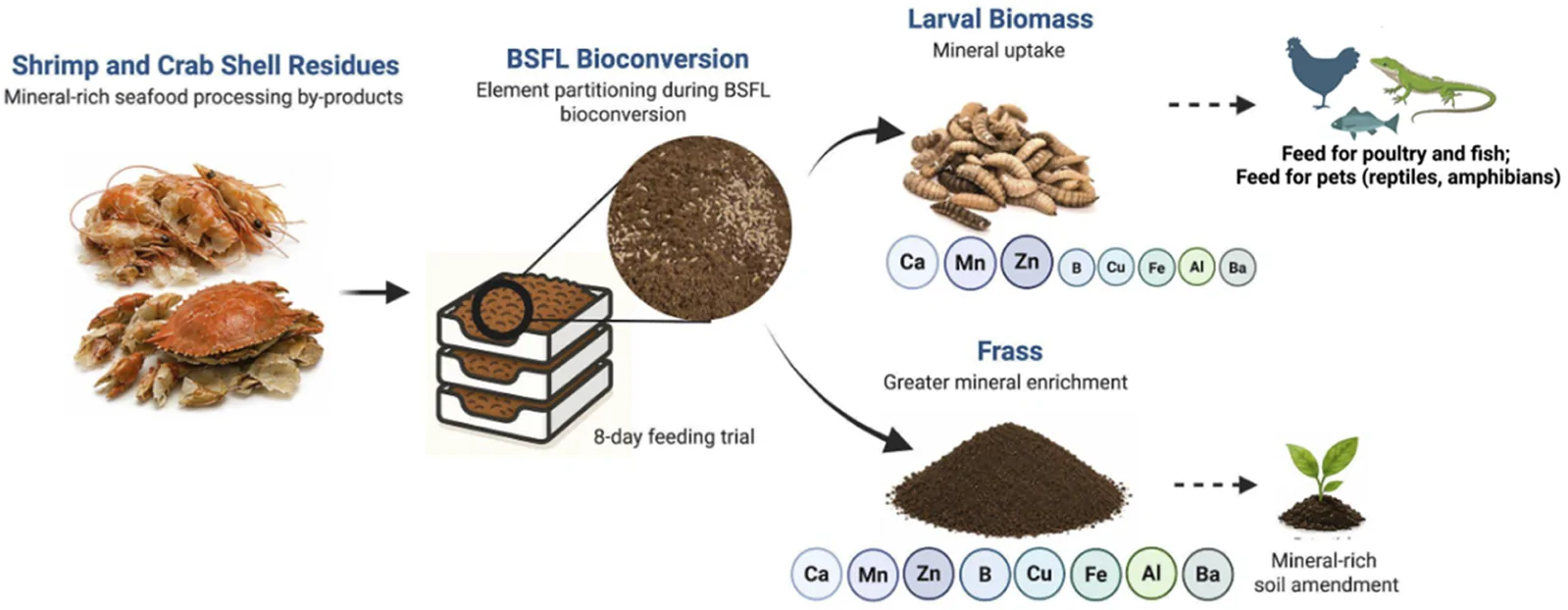

## Introduction

The global organic waste generation is rapidly rising each year, creating an urgent need for sustainable disposal and recycling solutions.^1^ Roughly one-third of all food produced for human consumption is lost or wasted each year, amounting to more than one billion tons globally.^2^ This growing waste issue has driven interest in innovative approaches like insect-mediated bioconversion for waste management. In particular, the black soldier fly (BSF, *Hermetia illucens*) has emerged as a highly effective tool for organic waste valorisation.^3,4^ The bioconversion process significantly reduces waste volume and, at the same time, recirculates nutrients back into the production cycle. Research shows that the larvae can reduce the volume of organic waste up to 80% (ref. 3) within two weeks, reducing the load on landfills and protecting the ecosystem from the release of harmful gases as the waste decomposes.^5^ Driven by its multiple benefits, there has been growing interest as well as growth in the insect industries worldwide.^6^ Many substrates contain substantial mineral content, such as calcium (Ca), phosphorus (P), potassium (K), as well as trace metals, including both essential micronutrients iron (Fe), zinc (Zn), copper (Cu) and heavy metals (cadmium (Cd), lead (Pb), mercury (Hg)).^7,8^ When BSF larvae feed on these wastes, these elements end up in different outputs: the insect biomass (larvae/prepupae), the residual frass, or, depending upon their chemical properties, may also volatilize. However, as this industry scales up to process millions of tons of waste, one critical question arises: what happens to the chemical constituents in particular minerals and trace metals that are present in the waste, and how do they partition into the outputs of bioconversion? Recently, research has begun addressing these questions, showing that substrate composition, metal speciation, and larval physiology all play roles in metal partitioning.^9–11^ However, data on certain waste streams and metal species are limited.

The chemical mechanisms of metal binding in insect exoskeletons and insect-derived biomaterials such as frass are primarily driven by two major structures: chitin and calcium carbonate. Chitin is a linear polysaccharide composed of repeating *N*-acetyl-d-glucosamine units linked with β-(1→4) glycosidic bonds. This structure contains hydroxyl and acetamide functional groups, which can offer electron donor sites for coordinated complexes with divalent and trivalent metal ions, including Cu^2+^, Zn^2+^, Cd^2+^, Pb^2+^, Ni^2+^, and Fe^3+^.^12,13^ Though chitin has a lower metal-binding capacity, its deacetylated derivative, chitosan, has demonstrated higher affinity for heavy metal sorption and is produced during specific growth cycles, insect defense stages, or bacterial conversion.^14,15^ This absorption mechanism is strongly influenced by pH, where lower pH will reduce active binding sites, while deprotonation at neutral or slightly alkaline pH enhances metal complex formation with an affinity to oxygen-containing functional groups as the dominant binding mechanism for Pb, Cu, Ni, Cd, and Zn to chitin.^16^ Within the chitin-protein matrix, amorphous calcium carbonate can also add additional pathways for metal binding. Carbonate-rich environments support higher metal-carbonate complexes, thus reducing dissolved metal concentrations and bioavailability. This mineral sequestration mechanism occurs when the Ca^2+^ in the crystal lattice is replaced by divalent cations such as Cd^2+^, Sr^2+^, Pb^2+^, Zn^2+^, and Mn^2+^.^17^ Metals can also adsorb directly to mineral surfaces and precipitate in soluble carbonate phases.^17^

Before forming complexes or being excreted in frass, the bioavailability of metals from substrates is influenced by larval gut conditions. The digestive tract has various pH gradients, enzymes, increased microbial activity, and substrate dissolved organic matter, which can alter mechanisms for metal accumulation and excretion.^18^ For instance, under alkaline conditions with varying pH and redox conditions, metals have an affinity to form complexes with carbonate or chitin-associated hydroxide functional groups.^18,19^ This reduces the overall free metal complexes and promotes precipitation. Organic ligands that are produced during digestion have an alternative effect where the bound metals maintain solubility and are available in the frass after excretion.^18,19^ Metals can also be chelated or mediate redox reactions in the digestive tract as organic acids are produced. In many cases, the substrate metal composition is a critical factor in understanding and assessing the safety of insect-based waste for circular bio-economy applications.^20^

The optimal mineral and metal composition in feedstocks are shaped by species-specific metabolic needs. In agriculture, a variety of species, including poultry, cattle, swine, and fish, have different demands for Zn, Cu, Fe, manganese (Mn), and selenium (Se) for immune function, growth, gut microbial activity, and development. For example, poultry focuses on two types of growth for broilers and layers. Broilers are grown for meat and have standardized nutritional requirements focusing on P and K to support growth, while layers have increased Ca, Mn, and Se for improved egg quality.^21,22^ Similarly, the cattle industry is subdivided into specific needs for beef and dairy production. Though there is minimal need for minerals and metals in dry matter forage, beef cattle have lower demand for Ca and P with a high Cu sensitivity.^23^ Dairy cattle have diets with high Ca, P, and Mg, while Se and Zn are more regulated due to the impact on mastitis.^24^ Swine agriculture focuses on piglet growth, gut health, and carcass traits for optimal sizing, where Ca, P, and Fe are vital for skeletal development, tissue deposition, and haemoglobin synthesis. Additional compounds like Zn and Cu are important for immune function but can be harmful at concentrations above the standard nutritional requirement of 100 ppm and 15 ppm, respectively, due to the reduced digestibility.^25,26^ Many research studies have shown the benefit of using BSF larvae as a protein supplement for terrestrial-based livestock, but there has been little focus on subcategories of nutrients from mineral and metal concentrations in larvae from initial waste streams.^27–29^ Aquaculture feedstock for farm-raised fish has taken more active approaches to tracing micronutrients for fish development, as BSF larvae can provide fully supported diets. The absorption and accumulation concerns in farm-raised fish have led to studies that show optimizing the diet blend to reduce overexposure to certain compounds.^30,31^ For instance, there is a strong correlation between feed-to-fish ratios for Mn, Zn, and Cu that may lead to bioaccumulation from feed to fish muscle.^32^ As BSF larvae continue to grow in popularity as a partial and full feed option, it is critical to trace elemental compound fate in larvae to ensure safe potential as feedstock.

For plant-based systems, mineral and metal concentrations from fertilizers are also a critical component for developing crops. BSF frass can be a beneficial additive, but nutrient demand is highly dependent on the agricultural commodity, and with the right blend can significantly reduce the amendments needed by producers. For instance, maize is highly susceptible to zinc deficiency, and without zinc enrichment, it can decrease yield due to insufficient root growth.^33^ Soybean is another agricultural row crop that has a higher concentration of Fe and Mn for optimal chlorophyll synthesis, photosynthesis, and nitrogen fixation.^34^ There are unique cropping systems like alfalfa and clover that are used for forage crops, which require a high demand for boron to aid in cell wall structure and flowering.^35^ Overall, large-scale applications of BSF frass may not be sustainable, based on current insect-rearing operations, but other greenhouse and horticulture industries can benefit from mixed substrate blends. An example is tomatoes, which benefit from Ca, Fe, and Mn additions, which maximize yields and fruit quality, while cucumbers are prone to Zn and Cu deficiency that stunt growth.^36,37^ Other vegetables, like carrots, cabbage, and greens, can act as “hyperaccumulators” and are highly sensitive to metal toxicity, which requires strict application to ensure balanced substrates.^36,38^ In both row crop agriculture and greenhouse nursery substrates, the introduction of vital elemental compounds is a balance between species and nutritional requirements. The current landscape of insect frass as fertilizer focuses primarily on the macronutrients, while insight into the bioaccumulation of minerals and metals is critical for cropping systems.

There have been numerous explorations on different types of organic waste streams in relation to their feasibility as a larval feed substrate.^39–41^ Yet, there is a gap in knowledge regarding the use of seafood processing by-products, mainly shrimp and crab shell residues, as rearing substrates. Crustacean shell wastes are rich in chitin and minerals (particularly calcium carbonate)^42^ and may contain a range of trace elements. Despite their abundance, the fate of these nutrients and potential contaminants during BSF processing is not well understood. Few studies have explored BSF larvae feeding on shrimp or crab shell waste,^43,44^ and none have examined mineral and metal partitioning across feed, larvae, and frass in these systems. This study addresses that gap by quantifying the flows of mineral and trace metals in a BSF bioconversion system using shrimp and crab shell residues. Specifically, we measure element concentrations in the input feed, larval biomass, and resulting frass to determine how these elements accumulate, transform, or are excreted. By understanding these mineral and metal flows, our work provides insights into the micronutrient cycling efficiency and safety of BSF-mediated seafood waste valorisation.

## Experimental

### Study site

Black Soldier Fly Larvae (BSFL) feeding trial was conducted at Fluker's Cricket Farm (Port Allen, Louisiana, USA), a commercial insect production facility. The facility provided controlled rearing conditions suitable for large-scale BSFL cultivation. All experimental diets were prepared and reared on site using commercial production trays, and BSFL used in the study were sourced from the farm's in-house colony. Previously published work details experimental design and protocols related to seafood waste studies.^45,46^ The trial was designed to evaluate mineral and trace element distribution among feed substrates, larval biomass, and residual frass under macro-scale rearing conditions to mitigate the uncertainty of benchtop trials.

### Diet formulation

Shrimp shell and crab shell residues were sourced from local Louisiana seafood processors. Shells were defined as the remaining exoskeletal material after removal of marketable meat. Seafood waste was ground using a Robot Coupe® R23 Series A vertical cutter mixer (Robot-Coupe U.S.A., Inc., Ridgeland, MS, USA), with tap water added incrementally to facilitate homogenization. The resulting paste was stored in food-safe containers in a refrigerator at 4 °C. Shrimp and crab shells were processed separately and incorporated into a commercial grain-based insect feed (commercial control; CC) at four inclusion levels: 25%, 40%, 50%, and 60% (w/w). A 100% commercial feed diet served as a positive control. Diet formulations were adjusted with water to maintain comparable substrate moisture across treatments. Each replicate tray received approximately 20 000 five-day-old BSF larvae to perform neonate inoculation diet incorporation trials. By introducing neonate larvae, this mimics industrial insect rearing conditions without inducing starvation from withheld substrate. All treatments were reared for eight days under controlled temperature (29.6 ± 0.9 °C) and humidity (76.5 ± 2.6%) in large-scale production trays. Shrimp- and crab-based trials were conducted independently, each with identical formulation ratios but separate control groups. Each treatment group was replicated three times.

At harvest, larvae were mechanically separated from frass using standard 8-mesh and 10-mesh sieves. Representative samples of initial feed mixtures (shrimp-based or crab-based diets), harvested larvae, and residual frass were collected from each treatment replicate during the macro trial (Fig. 1). All samples were homogenized manually prior to subsampling. Feed samples were collected immediately after diet formulation, while frass samples were collected following the eight-day trial period. The larvae were harvested after a 48-hour gut-clearance step to ensure all frass was evacuated. Subsamples intended for mineral analysis were stored at −20 °C until processing.

**Fig. 1:**
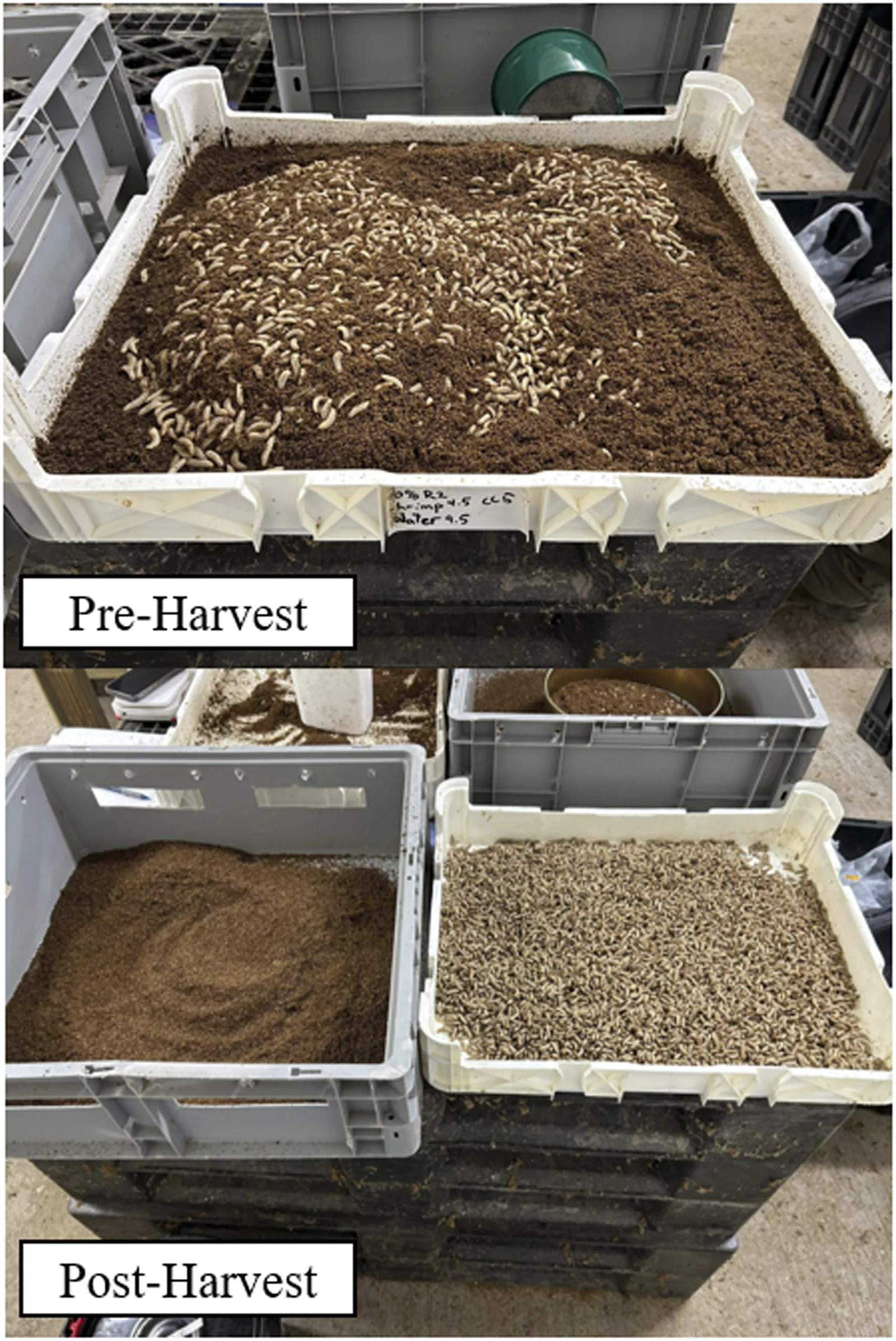
Pre-harvest larvae and frass mixed illustrating the organic matter produced from seafood waste, while post-harvest separation shows independent larvae and frass samples that were homogenized and subsampled for analysis.

### Mineral and trace element analysis

Elemental analyses were performed at the Agricultural Chemistry Laboratory, Louisiana State University. The Department of Agricultural Chemistry provides analytical support for both university and the state Department of Agriculture and Forestry. By contracting this service lab, the external analysis was completed without bias and under precision analytical techniques with standard instrument validations, calibrations, and methods. Feed, larval biomass, and frass samples were analysed for selected macro- and trace elements using inductively coupled plasma optical emission spectroscopy (ICP-OES). Feed, larval biomass, and frass samples were analysed for selected macro- and trace elements using inductively coupled plasma optical emission spectroscopy (ICP-OES). The elements quantified and discussed in this study included boron (B), calcium (Ca), iron (Fe), manganese, zinc (Zn), aluminium (Al), and barium (Ba). These elements were selected to evaluate mineral partitioning behaviour between feed substrates, larval biomass, and residual frass. Additional compounds, cadmium, chromium, cobalt, lead, molybdenum, nickel, selenium, and arsenic, were also analysed by the Agricultural Chemistry Laboratory and reported for seafood waste substrates, but yielded low concentrations with insignificant trends during the trials and thus were not discussed in this manuscript (see Tables S1–S6). Analyses were conducted following EPA Method 200.7,^47^ AOAC Method 985.01,^48^ and EPA Method 3052,^49^ as appropriate for each element. This includes freeze drying all analysed samples at temperatures below −40 °C prior to instrumental extraction methods. All concentrations reported in this study are expressed on a dry matter basis unless otherwise noted.

### Partitioning calculations and statistical analysis

To evaluate mineral and trace element partitioning, enrichment ratios were calculated separately for larvae and frass. All parameters were calculated on a dry matter basis to allow consistent comparison across diets with different moisture contents.^46^ The selected substance was divided by its corresponding feed concentrations to determine the increased ratio relative to diet blends. An enrichment ratio greater than one indicates higher element concentration in larvae or frass relative to feed, whereas values below one indicates lower concentrations relative to feed. Enrichment ratios were calculated independently for shrimp-based and crab-based trials to avoid cross-substrate comparison bias. Element concentration data are presented as mean ± SD from three independent biological replicates per treatment. Statistical analyses were performed using R (version 4.3). Treatment effects were evaluated using one-way analysis of variance within each trial (shrimp or crab). Normality and homogeneity of variance were assessed prior to ANOVA, and Tukey's HSD *post hoc* test was applied for multiple comparisons when significant treatment effects were detected. Statistical significance was assessed at *α* = 0.05. Heatmaps were generated in R to visualize enrichment patterns across treatments and matrices (larvae *vs.* frass). Data matrices of enrichment ratios were scaled by row to facilitate comparison of relative partitioning trends among treatments within each element. Heatmaps were produced using the ggplot2 package following data reshaping with tidyr, and color gradients were selected to highlight relative enrichment and depletion. In addition to the within-trial one-way ANOVA, an ordinary two-way ANOVA was performed using GraphPad Prism (version 11.0.2) for the shell-amended treatments (25%, 40%, 50%, and 60% inclusion). Substrate type (shrimp or crab shell), inclusion level, and their interaction were evaluated. Each substrate × inclusion combination included three independent production trays. The 100CC controls were excluded because separate control batches were used for the shrimp and crab trials. Tukey's multiple comparisons test was used to compare inclusion levels within each substrate. For all statistical analyses, significance was set at *α* = 0.05.

## Results and discussion

### Substrate mineral and trace element composition

The shrimp and crab shell waste differed in their mineral and trace metal profile (Table 1). Crab shells contained higher concentrations of calcium (20.29 ± 0.05%) compared to shrimp shells (16.06 ± 0.91%). Crab shells also contained elevated manganese and barium concentrations (440.46 ± 116.40 ppm Mn; 375.01 ± 85.71 ppm Ba), whereas shrimp shells showed comparatively higher boron (39.74 ± 1.30 ppm), aluminium (164.05 ± 103.16), and zinc (30.50 ± 2.23). Iron and copper concentrations were similar between shrimp and crab shells.

**Table 1:** Mineral and trace element composition of shrimp and crab shell substrates reported on dry matter basis

Parameters	Shrimp shell (mean ± SD)	Crab shell (mean ± SD)
Boron (PPM)	39.74 ± 1.30	15.66 ± 0.25
Calcium %	16.06 ± 0.91	20.29 ± 0.05
Copper (PPM)	7.15 ± 4.86	7.53 ± 3.75
Iron (PPM)	90.94 ± 31.09	92.14 ± 17.52
Manganese (PPM)	31.92 ± 6.37	440.46 ± 116.40
Zinc (PPM)	30.50 ± 2.23	24.44 ± 6.10
Aluminium (PPM)	164.05 ± 103.16	91.13 ± 25.09
Barium (PPM)	64.39 ± 11.76	375.01 ± 85.71
Molybdenum (PPM)	1.40 ± 0.02	0.78 ± 0.01
Selenium (PPM)	11.84 ± 0.30	7.83 ± 0.12
Cobalt (PPM)	0.59 ± 0.02	0.39 ± 0.01
Arsenic (PPM)	5.92 ± 0.15	3.91 ± 0.06
Nickel (PPM)	0.97 ± 0.15	0.59 ± 0.01
Lead (PPM)	3.44 ± 2.55	3.59 ± 0.41
Chromium (PPM)	2.37 ± 0.53	3.69 ± 1.80
Cadmium (PPM)	0.81 ± 0.01	0.59 ± 0.01

### Elemental composition of feed formulations

Within the shrimp-based trial, increasing inclusion of shrimp shell residue resulted in changes in feed mineral composition compared to the shrimp trial control (100CC) (Table 2). Calcium concentrations increased significantly with shell inclusion, rising from 1.06 ± 0.04% in the control to a maximum of 3.51 ± 0.20% in the 40S60CC formulation. Barium concentrations also increased across formulations, ranging from 8.09 ± 1.46 ppm in the control to 20.30 ± 1.21 ppm in the 40S60CC treatment. Manganese and zinc concentrations varied significantly among formulations, with the highest values observed in the 25S75CC and 60S40CC treatments, respectively. Copper concentrations differed significantly across treatments, while aluminium concentrations did not vary significantly among shrimp-based feed formulations. Iron concentrations remained within a narrow range across all treatments.

**Table 2:** Elemental composition of shrimp-based feed formulations reported on dry matter basis

Parameters	100CC (mean ± SD)	25S75CC (mean ± SD)	40S60CC (mean ± SD)	50S50CC (mean ± SD)	60S40CC (mean ± SD)
Boron (PPM)	49.18 ± 8.63^a^	25.30 ± 1.24^a^	33.36 ± 2.85^a^	37.04 ± 10.36^a^	27.81 ± 18.61^a^
Calcium %	1.06 ± 0.04^d^	2.03 ± 0.12^c^	3.51 ± 0.20^a^	3.00 ± 0.05^b^	2.98 ± 0.21^b^
Copper (PPM)	31.23 ± 2.12^b^	39.31 ± 0.53^a^	25.80 ± 0.76^c^	26.97 ± 0.30^c^	37.46 ± 1.25^a^
Iron (PPM)	144.35 ± 1.49^ab^	152.97 ± 7.10^a^	133.05 ± 1.73^b^	140.20 ± 6.80^ab^	154.35 ± 6.35^a^
Manganese (PPM)	79.37 ± 1.96^b^	100.48 ± 1.03^a^	70.92 ± 2.31^c^	74.73 ± 1.56^bc^	100.54 ± 4.37^a^
Zinc (PPM)	121.94 ± 2.02^c^	150.31 ± 2.44^a^	102.62 ± 1.67^d^	106.00 ± 0.94^d^	141.70 ± 3.63^b^
Aluminium (PPM)	64.33 ± 2.30^a^	73.80 ± 10.74^a^	82.88 ± 3.06^a^	100.85 ± 34.79^a^	76.70 ± 5.06^a^
Barium (PPM)	8.09 ± 1.46^d^	12.39 ± 0.45^c^	20.30 ± 1.21^a^	18.13 ± 1.24^ab^	17.49 ± 0.18^b^

Crab-based feed formulations exhibited pronounced changes in mineral and trace element concentrations relative to the crab trial control (100CC) (Table 3). Calcium concentration increased progressively with crab shell inclusion, from 1.11 ± 0.06% in the control to 9.56 ± 0.30% in the 60C40CC formulation. Manganese and barium concentrations showed strong monotonic increases with shell inclusion, reaching 223.68 ± 3.47 ppm Mn and 133.74 ± 10.95 ppm Ba in the highest inclusion treatment.

**Table 3:** Elemental composition of crab-based feed formulations reported on dry matter basis

Parameters	100CC (mean ± SD)	25C75CC (mean ± SD)	40C60CC (mean ± SD)	50C50CC (mean ± SD)	60C40CC (mean ± SD)
Boron (PPM)	26.31 ± 6.62^a^	23.19 ± 2.28^a^	24.81 ± 0.88^a^	28.06 ± 1.08^a^	28.40 ± 1.65^a^
Calcium %	1.11 ± 0.06^e^	4.06 ± 0.19^d^	6.08 ± 0.20^c^	7.03 ± 0.28^b^	9.56 ± 0.30^a^
Copper (PPM)	34.18 ± 1.47^a^	24.56 ± 2.63^cd^	21.98 ± 1.05^d^	30.64 ± 2.87^ab^	28.29 ± 0.58^bc^
Iron (PPM)	275.62 ± 5.97^a^	269.81 ± 18.33^ab^	237.02 ± 19.37^bc^	241.71 ± 7.72^abc^	229.42 ± 4.29^c^
Manganese (PPM)	108.28 ± 1.63^d^	151.47 ± 4.25^c^	174.95 ± 8.25^b^	190.47 ± 9.38^b^	223.68 ± 3.47^a^
Zinc (PPM)	134.58 ± 3.83^a^	115.23 ± 10.97^b^	100.29 ± 3.14^c^	99.37 ± 1.60^c^	89.01 ± 1.82^c^
Aluminium (PPM)	103.32 ± 4.93^c^	113.68 ± 5.28^bc^	115.08 ± 13.00^bc^	131.89 ± 5.29^ab^	141.43 ± 4.44^a^
Barium (PPM)	8.62 ± 0.37^d^	57.94 ± 1.97^c^	88.08 ± 3.98^b^	101.85 ± 5.96^b^	133.74 ± 10.95^a^

Zinc concentrations decreased with increasing crab shell inclusion, while aluminium concentrations increased across formulations. Iron concentrations declined steadily from the control to higher shell inclusion treatments. Copper concentrations varied significantly among treatments but did not follow a monotonic trend.

### Elemental composition of larvae reared on seafood waste

Larvae reared on shrimp-based diets showed significant differences in elemental composition across treatments (Table 4). Larval calcium concentrations were higher in shell-amended diets compared to the control, with values ranging from 2.56 ± 0.30% (control) to 3.73 ± 0.22% (40S60CC). Boron concentrations varied modestly among treatments, while iron concentrations remained statistically similar across all formulations. Manganese concentrations declined with increasing shell inclusion beyond the 25S75CC treatment, decreasing from 207.47 ± 10.69 ppm to 60.74 ± 6.68 ppm. Zinc concentrations peaked in larvae reared on the 40S60CC diet (191.32 ± 20.40 ppm). Aluminium concentrations increased at higher inclusion levels, reaching 71.41 ± 4.47 ppm in the 60S40CC treatment. Barium concentrations varied significantly but remained within a narrow range across treatments.

**Table 4:** Elemental composition of larvae reared on shrimp-based diets reported on dry matter basis

Parameters	100CC (mean ± SD)	25S75CC (mean ± SD)	40S60CC (mean ± SD)	50S50CC (mean ± SD)	60S40CC (mean ± SD)
Boron (PPM)	16.35 ± 2.80^ab^	15.58 ± 0.52^ab^	19.06 ± 0.26^a^	14.53 ± 1.06^b^	19.01 ± 0.89^a^
Calcium %	2.56 ± 0.30^b^	3.54 ± 0.20^a^	3.73 ± 0.22^a^	3.17 ± 0.42^ab^	3.27 ± 0.24^ab^
Copper (PPM)	22.39 ± 2.78^b^	31.40 ± 5.79^a^	23.95 ± 1.95^ab^	16.48 ± 1.84^b^	22.89 ± 1.19^b^
Iron (PPM)	134.55 ± 5.27^a^	126.51 ± 6.91^a^	129.67 ± 11.88^a^	117.62 ± 8.30^a^	126.07 ± 5.10^a^
Manganese (PPM)	191.85 ± 21.26^a^	207.47 ± 10.69^a^	118.00 ± 14.70^b^	74.44 ± 9.50^c^	60.74 ± 6.68^c^
Zinc (PPM)	128.84 ± 8.20^b^	151.51 ± 5.87^b^	191.32 ± 20.40^a^	140.33 ± 16.11^b^	127.89 ± 10.09^b^
Aluminium (PPM)	36.21 ± 0.17^b^	33.67 ± 3.70^b^	45.92 ± 4.91^b^	40.47 ± 7.21^b^	71.41 ± 4.47^a^
Barium (PPM)	10.09 ± 0.93^c^	13.44 ± 0.62^b^	17.30 ± 0.75^a^	14.19 ± 2.02^b^	15.25 ± 0.82^ab^

In the crab-based trial, larval calcium concentrations were significantly higher in shell-amended diets compared to the control, with values ranging from 4.75 ± 0.27% to 5.65 ± 0.59% (Table 5). Iron concentrations decreased significantly with increasing shell inclusion, from 243.88 ± 14.36 ppm in the control to approximately 136–147 ppm in higher inclusion treatments. Manganese concentrations declined sharply relative to the control, while zinc concentrations decreased progressively with increasing shell inclusion. Aluminium concentrations increased across treatments, reaching 84.20 ± 5.79 ppm in the 60C40CC formulation. Barium concentrations were significantly elevated in all shell-amended diets relative to the control.

**Table 5:** Elemental composition of larvae reared on crab-based diets reported on dry matter basis

Parameters	100CC (mean ± SD)	25C75CC (mean ± SD)	40C60CC (mean ± SD)	50C50CC (mean ± SD)	60C40CC (mean ± SD)
Boron (PPM)	17.97 ± 1.93^a^	16.51 ± 0.66^a^	18.53 ± 4.40^a^	17.26 ± 2.21^a^	16.92 ± 1.68^a^
Calcium %	2.60 ± 0.04^b^	5.56 ± 0.44^a^	5.65 ± 0.59^a^	4.75 ± 0.27^a^	5.44 ± 0.52^a^
Copper (PPM)	26.11 ± 1.57^ab^	25.73 ± 1.54^ab^	28.68 ± 2.01^a^	23.24 ± 1.80^b^	23.12 ± 2.76^b^
Iron (PPM)	243.88 ± 14.36^a^	189.71 ± 34.70^b^	146.68 ± 9.76^bc^	136.37 ± 11.37^c^	146.90 ± 10.96^bc^
Manganese (PPM)	273.90 ± 14.47^a^	221.21 ± 19.70^b^	130.94 ± 15.82^c^	102.05 ± 9.14^c^	108.21 ± 11.51^c^
Zinc (PPM)	176.37 ± 8.02^a^	150.91 ± 12.82^a^	107.96 ± 11.48^b^	89.20 ± 9.22^b^	86.88 ± 7.74^b^
Aluminium (PPM)	51.95 ± 3.90^c^	54.90 ± 7.35^c^	72.39 ± 5.27^ab^	66.16 ± 3.32^bc^	84.20 ± 5.79^a^
Barium (PPM)	12.05 ± 2.11^b^	36.82 ± 1.56^a^	37.89 ± 5.94^a^	30.57 ± 2.34^a^	39.42 ± 4.99^a^

### Elemental composition of frass produced from seafood waste

Frass derived from shrimp-based diets showed substantial enrichment of several elements relative to corresponding feed formulations (Table 6). Calcium concentrations increased significantly with shell inclusion, reaching 7.34 ± 0.30% in the 60S40CC treatment. Manganese concentrations were significantly higher in frass from all shell-amended diets compared to the control, whereas iron and zinc showed treatment-dependent responses. Aluminium concentrations did not differ significantly among treatments, while barium concentrations increased progressively with shell inclusion, reaching 42.62 ± 2.84 ppm in the highest inclusion treatment. Boron concentrations did not vary significantly across shrimp-based frass samples.

**Table 6:** Elemental composition of frass from shrimp-based diets reported on dry matter basis

Parameter	100CC (mean ± SD)	25S75CC (mean ± SD)	40S60CC (mean ± SD)	50S50CC (mean ± SD)	60S40CC (mean ± SD)
Boron (PPM)	33.60 ± 12.00^a^	50.72 ± 6.18^a^	46.76 ± 10.03^a^	37.50 ± 3.87^a^	31.42 ± 2.01^a^
Calcium %	1.42 ± 0.06^d^	3.96 ± 0.39^c^	6.28 ± 0.15^ab^	5.26 ± 0.72^b^	7.34 ± 0.30^a^
Copper (PPM)	58.42 ± 6.75^b^	82.81 ± 5.71^a^	81.51 ± 3.59^a^	53.68 ± 3.34^b^	52.59 ± 1.12^b^
Iron (PPM)	301.81 ± 33.96^b^	364.61 ± 18.22^ab^	395.63 ± 19.66^a^	320.23 ± 27.41^b^	338.94 ± 27.40^ab^
Manganese (PPM)	74.27 ± 6.45^c^	145.74 ± 7.86^b^	214.60 ± 9.64^a^	137.66 ± 17.18^b^	147.82 ± 10.66^b^
Zinc (PPM)	214.37 ± 19.30^b^	301.66 ± 25.67^a^	254.34 ± 17.99^b^	159.03 ± 4.41^c^	153.64 ± 11.11^c^
Aluminium (PPM)	151.56 ± 25.67^a^	200.10 ± 30.63^a^	205.94 ± 5.85^a^	185.74 ± 18.58^a^	197.23 ± 19.16^a^
Barium (PPM)	12.36 ± 1.37^d^	21.52 ± 2.54^c^	30.35 ± 0.94^b^	29.64 ± 3.71^b^	62. ±2.84^a^

Frass from crab-based diets exhibited strong, treatment-dependent changes in mineral composition (Table 7). Calcium concentrations increased monotonically with shell inclusion, ranging from 1.74 ± 0.03% in the control to 12.15 ± 0.23% in the 60C40CC treatment. Iron concentrations decreased significantly across treatments, while manganese concentrations increased, exceeding 275 ppm in higher inclusion formulations. Zinc concentrations declined progressively with shell inclusion, whereas aluminium concentrations remained relatively stable across treatments. Barium concentrations increased substantially with crab shell inclusion, reaching 149.88 ± 13.21 ppm in the highest inclusion treatment. Boron concentrations decreased relative to the control.

**Table 7:** Elemental composition of frass from crab-based diets reported on dry matter basis

Parameters	100CC (mean ± SD)	25C75CC (mean ± SD)	40C60CC (mean ± SD)	50C50CC (mean ± SD)	60C40CC (mean ± SD)
Boron (PPM)	46.45 ± 1.15^a^	43.17 ± 2.79^a^	34.77 ± 4.23^b^	33.62 ± 3.25^b^	31.33 ± 1.46^b^
Calcium %	1.74 ± 0.03^e^	5.30 ± 0.12^d^	8.47 ± 0.40^c^	9.50 ± 0.21^b^	12.15 ± 0.23^a^
Copper (PPM)	70.96 ± 1.19^a^	68.77 ± 5.47^a^	52.80 ± 1.47^b^	51.32 ± 2.22^b^	45.20 ± 1.83^b^
Iron (PPM)	635.73 ± 10.14^a^	603.92 ± 26.55^a^	507.28 ± 9.76^b^	444.90 ± 6.01^c^	374.99 ± 9.01^d^
Manganese (PPM)	167.27 ± 2.85^c^	214.76 ± 1.49^b^	275.31 ± 6.20^a^	272.83 ± 4.01^a^	287.82 ± 13.70^a^
Zinc (PPM)	283.78 ± 8.97^a^	247.68 ± 15.18^b^	204.30 ± 3.59^c^	180.70 ± 3.30^d^	145.17 ± 4.27^e^
Aluminium (PPM)	212.59 ± 1.80^b^	243.60 ± 13.99^a^	211.31 ± 10.94^b^	216.70 ± 12.41^ab^	205.63 ± 5.37^b^
Barium (PPM)	14.80 ± 0.36^d^	67.44 ± 4.39^c^	115.21 ± 1.27^b^	125.92 ± 8.65^b^	149.88 ± 13.21^a^

### Substrate and inclusion-level effects on element composition

Two-way ANOVA was used to test the effects of substrate type, inclusion level, and their interaction for the shell-amended treatments (Table S7). Substrate had a significant effect on calcium, iron, manganese, zinc, aluminium, and barium in both larvae and frass. Substrate also affected boron and copper in frass, while no significant substrate effect was detected for these two elements in larvae. Inclusion level was significant for all elements except aluminium in frass (*F* = 2.06, *p* = 0.146). The substrate × inclusion interaction differed between larvae and frass. In frass, significant interactions were detected for calcium, copper, iron, manganese, zinc, and barium (all *p* < 0.001), indicating that the effect of inclusion level depended on substrate type for these elements. In larvae, significant interactions were detected only for copper (*p* = 0.006) and zinc (*p* < 0.001). The interaction for larval iron was close to, but did not reach, the significance threshold (*p* = 0.0502).

### Enrichment patterns generated from seafood waste diets

In larvae, an enrichment ratio greater than 1 suggests bioaccumulation of elements, while a ratio less than 1 indicates the larvae are either consuming that element at a slower rate or it's being passed and accumulated in the waste. The optimal biological growth efficiency, where larvae convert the substrate into maximum body weight in the shortest amount of time, is represented by the 100CC which is the standard for industrial insect rearing. Across both seafood waste trials, calcium (Ca) in larvae exhibited the most consistent larval enrichment pattern. In larvae from control diets (100CC), Ca enrichment exceeded twofold in both shrimp and crab trials. With increasing shell inclusion, Ca enrichment declined in both datasets, approaching a ratio of 1 in shrimp-based diets and slowing consumption below optimal at higher crab shell inclusion levels. Manganese showed strong enrichment in larvae at lower shell inclusion levels in both trials, with enrichment ratios exceeding twofold in control diets and declining progressively with increasing shell inclusion. Zinc (Zn) exhibited moderate enrichment in larvae, with enrichment near or slightly above an enrichment ratio of 1 across treatments. In contrast, iron (Fe) remained near or below a ratio less than 1 across all treatments in both trials, indicating no substantial larval enrichment. Aluminium (Al) consistently showed enrichment ratios below one in larvae across shrimp and crab diets. Barium (Ba) exhibited limited larval enrichment, with ratios near 1 in shrimp diets, but declining at higher crab shell inclusion levels compared to 100CC (Fig. 2).

**Fig. 2:**
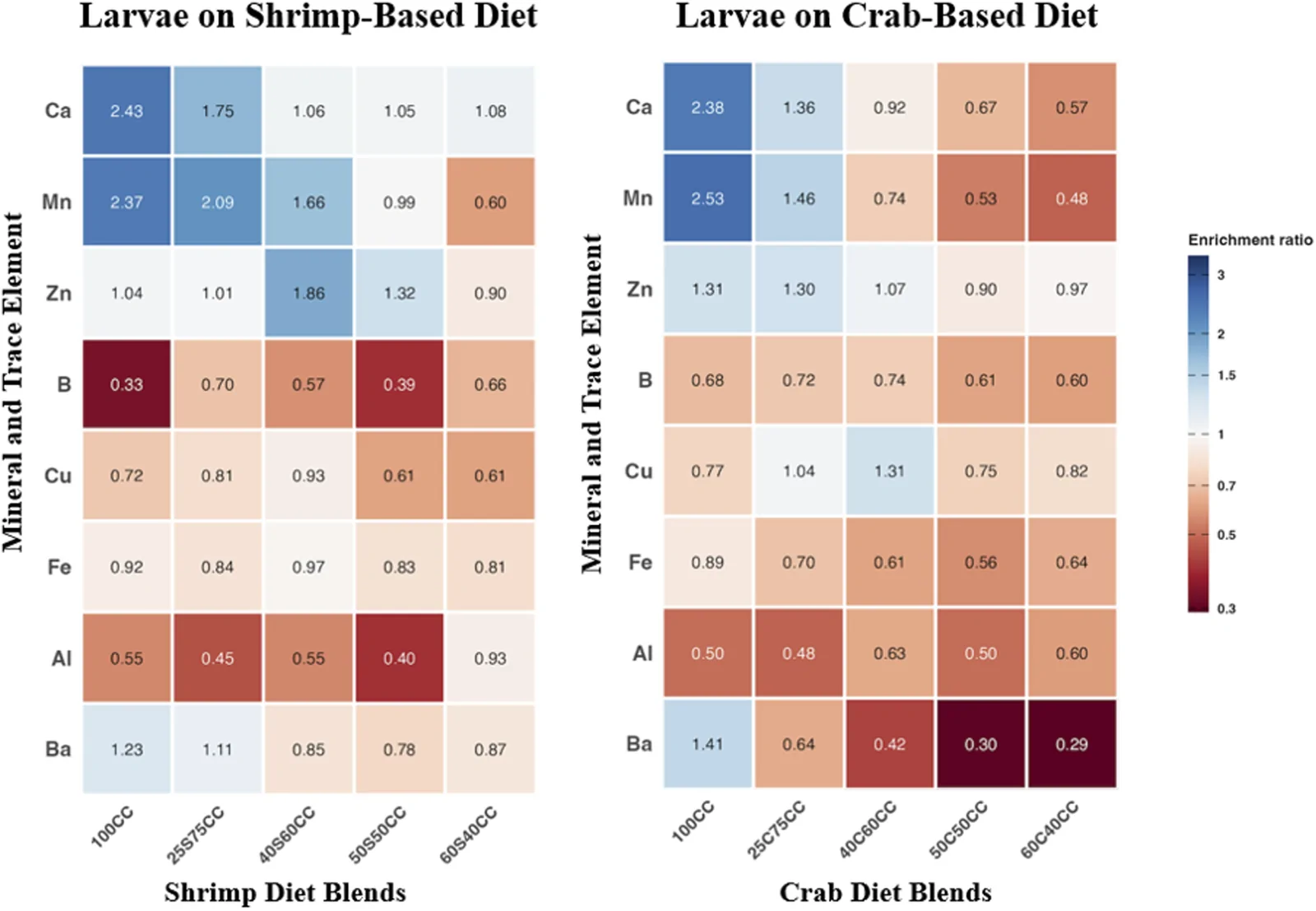
Enrichment density maps showing larvae-to-feed elemental ratios in black soldier fly larvae reared on shrimp-based (left) and crab-based (right) diets. Ratios greater than 1 suggest relative bioaccumulation in larvae, whereas ratio below 1 indicates slower consumption and concentration in waste.

In frass, an enrichment ratio greater than 1 indicates the concentration of the element in the waste. This occurs as the larvae digest large amounts of bulk matter and excrete the specific element at a much higher proportionate rate. In contracts, ratio below 1 suggests larval element absorption with a ratio of exactly 1 signifying no net accumulation or loss of the element. Enrichment ratios can be compared to the 100CC standard to measure the potential impact of seafood waste as an insect substrate. Frass consistently exhibited higher enrichment ratios than larvae for all reported elements in both trials. Calcium enrichment in frass increased with shrimp shell inclusion and remained elevated across crab-based diets. Iron showed strong and consistent enrichment in frass, with enrichment ratios exceeding twofold across most treatments in both trials. Copper (Cu) and zinc were consistently enriched in frass across shrimp and crab diets, with peak enrichment generally observed at intermediate shell inclusion levels. Aluminium demonstrated pronounced enrichment in frass across all treatments in both trials, with enrichment ratios consistently exceeding unity. Barium exhibited moderate frass enrichment in both datasets (Fig. 3).

**Fig. 3:**
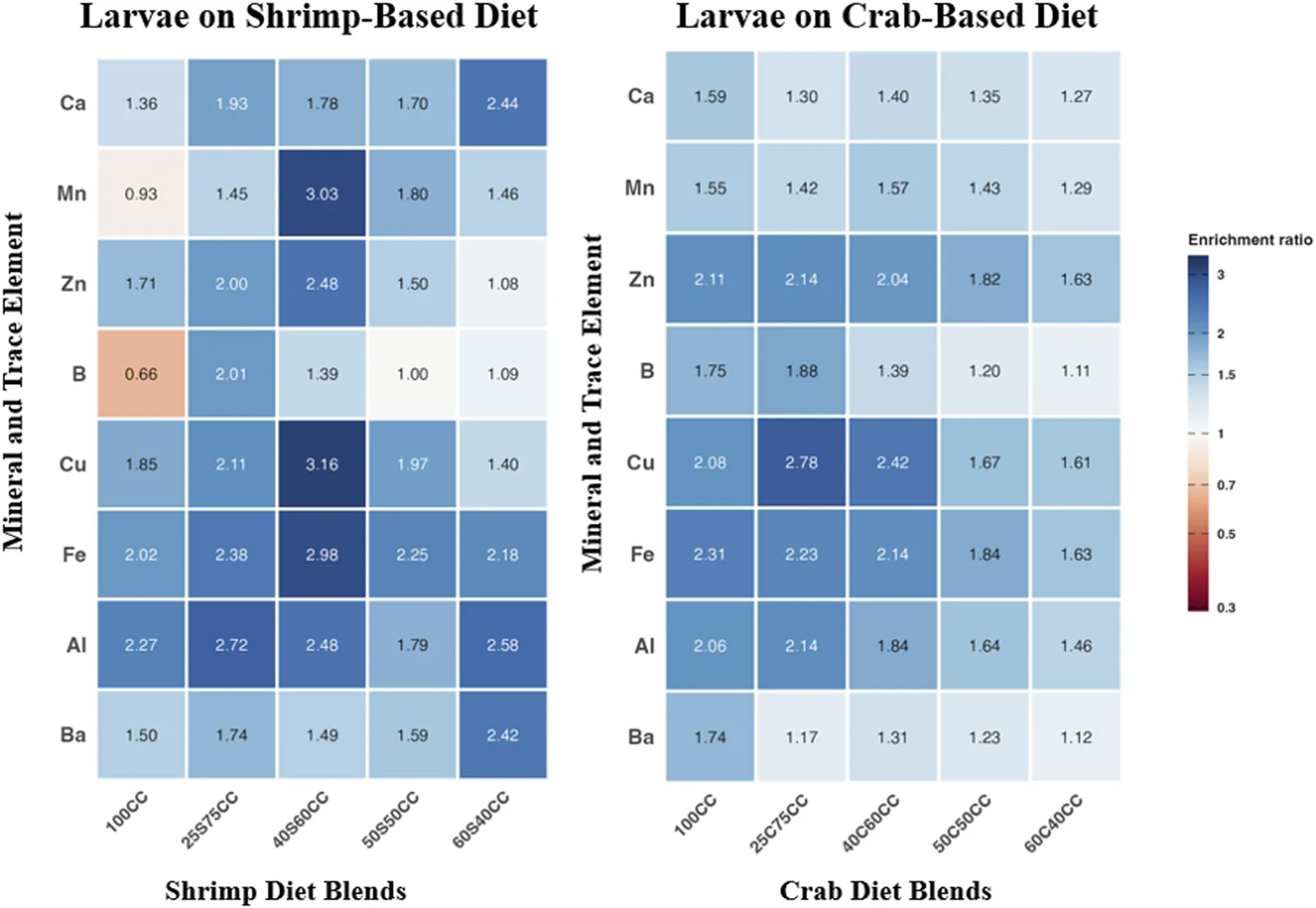
Enrichment density maps showing frass-to-feed elemental ratios in larvae waste from shrimp-based (left) and crab-based (right) diets. Ratios greater than 1 suggest relative concentration of the element in frass, whereas ratio below 1 indicates retention of the element by the larvae.

### Mineral partitioning between BSF larvae and frass

The present study revealed clear patterns of mineral partitioning between BSF larvae and frass when reared on crustacean-based diets. Across both shrimp- and crab-derived feeds, the bulk of certain minerals remained in the frass, while larvae assimilated only a fraction. Calcium (Ca) showed the most pronounced difference between larvae and frass. Larval Ca content increased only modestly with higher shell inclusion, especially in case of crab-based diet where the feed showed higher Ca content at higher shell inclusion levels. In contrast, frass Ca levels from both the shrimp and crab-based diets rose dramatically. Higher Ca in frass may reflect the presence of indigestible shell material, residual mineral matter, and limited larval assimilation capacity for excess Ca. This aligns with findings in other systems where BSF frass retains elevated Ca due to residual chitin and mineral matter.^50^

The partitioning of trace elements like iron, manganese, zinc, copper, aluminium, and barium differed between larvae and frass. These patterns depended on the substrate type and inclusion level. Mn and Zn showed moderate uptake into larvae, whereas Cu, Fe, B and Ba remained low in larvae but concentrated in frass. For instance, manganese was strongly enriched in larvae on low-shell diets (enrichment ratios between 1.5 to 2 in 100CC and 25% shell treatment groups). In crab-based diet, although Mn concentrations in feed remained high in higher shell diets, larval Mn dropped as with the increase in the shell inclusion in the diet. In the shrimp trial, larval Mn declined from ∼207 ppm at 25% shell inclusion to ∼61 ppm at the 60% shell diet. In the crab trial, the effect was even more pronounced: larval Mn plummeted from 221 ppm at 25% shell inclusion to ∼108 ppm at 60% shell inclusion. This inverse relationship in the crab-based diet is consistent with active regulation of essential trace elements in insects, a phenomenon commonly reported in BSF and other insect systems. This is largely due to the competitive inhibition of intestinal absorption in insect larvae, as both Ca and Mn use divalent, low-affinity transport channels. The saturation of Ca can block transport channels, which reduces the uptake and bioaccumulation of Mn.^51,52^ High Ca can also create alkaline gut conditions, which suppress the bioavailability of metals like Mn through mineralized complexes.^52^ When dietary availability is low, larvae increase uptake to meet metabolic demand, whereas excess dietary availability results in downregulated uptake.^53,54^ Correspondingly, frass Mn rose with shell content. In the shrimp-based frass, Mn increased more than 2-fold (from 74 ppm to 215 ppm) as shell inclusion went from 0% to 40%. Crab frass Mn also climbed with shell, reaching 280–288 ppm at the highest inclusion. The opposing trends observed in larvae and frass indicate that Mn retention by larvae was limited at higher dietary levels, resulting in greater Mn enrichment in the frass. While these patterns are consistent with selective uptake reported for BSF and other insects, the underlying physiological mechanisms were not directly assessed. The two-way ANOVA further showed a significant substrate × inclusion interaction for Mn in frass (*F*(3,16) = 26.41, *p* < 0.001), whereas the interaction was not significant in larvae (*p* = 0.108). This indicates that the frass Mn response to increasing shell inclusion differed between shrimp- and crab-based treatments.

Zinc (Zn) followed a pattern broadly similar to manganese, with reduced larval retention at higher shell inclusion levels and a corresponding enrichment of Zn in the frass across both shrimp- and crab-based diets. Fe, Al, and Ba were largely retained in the frass across all treatments. Unlike Mn, Zn, the larval concentrations of these elements remained low (below 1) across most of the treatment groups, whereas frass concentrations increased substantially. This indicates limited transfer of these elements into larval biomass during bioconversion.

Overall, frass consistently showed greater mineral enrichment than larvae across all elements examined. BSF larvae accumulated only a subset of dietary minerals, while the majority remained in the residual substrate. Although this study does not directly demonstrate physiological regulation, the observed, non-linear partitioning patterns across dietary gradients are consistent with selective mineral retention during BSF bioconversion. The outcomes support the use of BSF larvae for processing mineral-rich seafood waste while limiting undesirable mineral accumulation in larval biomass and concentrating nutrients in the frass.

### Impact of larvae bioaccumulations for feedstock

Potential uses of larvae as feedstock can be impactful outside the increase in protein content seen from shrimp and crab-based diets.^45,46^ Across both diet blends, Ca, Zn, and Mn have the most significant enrichment ratio in the larvae. Apart from 40S60CC Zn, each diet blend has the highest ratio in the control trial, whereas the inclusion of shells decreases suggesting no accumulation. Crab shell substrates have higher Ca and Mn content compared to shrimp shells, while Zn is similar in both diet blends (Tables 3 and 4). Standard nutritional diets for laying hens in the poultry industry include Ca: 3.5–4.5%, Mn: 90–120 mg kg^−1^, and Zn: 40–100 mg kg^−1^.^22,55^ For larvae reared on shrimp diets, Ca is optimal at lower shell inclusion, but Zn and Mn are higher than in standard diets. As Ca digestibility decreases with higher shrimp shell inclusion, Ca is lowered, while Mn and Zn remain slightly above standard nutritional diets for laying hens. For larvae raised on crab, 50% and 60% shell inclusion have optimal Zn and Mn with slightly elevated Ca for hen diets. In aquaculture, species of fish can dictate standard levels of micronutrients needed, Kokkali *et al.* 2025 identifies experimental diets for salmon to have concentrations of Fe (243–329 mg kg^−1^), Zn (163–208 mg kg^−1^), and Mn (68–82 mg kg^−1^), while Henry *et al.* 2020 shows optimal concentrations of Fe (100–300 mg kg^−1^) and Zn (50–150 mg kg^−1^) for sea bass.^56,57^ As seen, Zn and Mn concentrations are lower at higher shrimp and crab shell inclusion rates, while Fe is higher at lower inclusion rates, requiring a strategic decision when crafting aquaculture-based diets based on species needs. Our study yielded larvae with Fe concentrations of 126–134 mg kg^−1^ and 146–243 mg kg^−1^ in shrimp and crab, respectively, which could support experimental diets for sea bass, but would be under the threshold for salmon. A consideration for metal toxicity for non-essential metals like barium should be further explored as substrates are selected for feedstock trials. This study presents an increase larval barium concentration with 60% crab shell inclusion, the highest at ∼39 mg kg^−1^. Soluble barium is considered toxic in livestock feed at 40 mg kg^−1^ thresholds and should be monitored depending on the intended species.^56^ The total elemental barium reported in this study should be further analysed to determine soluble and insoluble forms for accurate feedstock safety recommendations. Studies have shown that total barium concentration ranges have varied impacts on anaerobic digestion as a feedstock.^58^ Overall, the selectivity of feedstock for specific types of livestock or aquaculture fish feed offers ample opportunities for continued research in diet formulation from diverse waste substrates.

### Nutrient recycling and agronomic potential of the frass

Our results show that BSF frass produced from shrimp and crab shells has potential as an organic soil amendment. These elemental concentrations do not directly equate to the plant-available fraction, but offer insight into the accumulation of metals that can be unlocked through organic acids and microbial activity in the soil. Future trials to determine the beneficial use of frass as amendments could include techniques such as X-ray diffraction or diethylenetriaminepentaacetic acid (DTPA) extractions to measure plant-available levels. Additional research can also be conducted using controlled greenhouse trials for ornamental plants and vegetables to see insoluble mineral response to acid soil substrates and direct plant availability. These initial results indicated frass from both substrates was enriched with several agronomically valuable nutrients, especially calcium. Calcium concentrations in the high inclusion shell diets ranged from approximately 7–12% (70–120 g kg^−1^), which were within the same range or exceeding the calcium content reported for common organic amendments such as chicken manure (∼16 g kg^−1^), dairy manure (∼72 g kg^−1^) and biochar (∼30 g kg^−1^).^59^ A calcium carbonate equivalent ratio can be used to estimate the amount of available Ca for liming, but it can be an overestimate.^17^ Insect frass consists of exchangeable Ca ions, organically bound Ca complexes, residual Ca carbonate, and Ca phosphates, making it difficult to determine exact concentrations for liming and fertilizer.^17,60^ For the most accurate interpretation, further titration methods are needed to determine the exact CaCO_3_ concentration. Because of the high CaCO_3_ content of crustacean shells, particularly crab shells, the resulting frass may function not only as a calcium fertilizer but also as a low-level liming agent, making it especially suitable for acidic or calcium-deficient soils. This shows a clear advantage of BSF bioconversion in transforming shell waste into a functional calcium-rich soil amendment. In addition to calcium, frass contained higher concentrations of essential micronutrients including zinc, manganese, copper and iron. Zinc and manganese concentrations in the frass (Zn: 145–300 mg kg^−1^, Mn: 145–287 mg kg^−1^) were comparable to those reported for dairy manure (Zn: 129 mg kg^−1^, Mn: 335 mg kg^−1^) and substantially higher than values reported for biochar (Zn: 22 mg kg^−1^, Mn: 90 mg kg^−1^).^61^ These micronutrients are needed in small amounts for plant growth and may act as slow-release nutrient sources when supplied through frass.^7^ Iron concentrations in the frass were lower than those reported for dairy manure and biochar;^61^ however, frass derived iron may still contribute to overall micronutrient inputs, particularly when applied as part of an integrated fertility strategy rather than as a standalone Fe amendment. The enrichment of zinc and manganese is particularly important for soils prone to deficiencies. Iron in the frass although not immediately bioavailable may become available over time through soil and microbial processes. Overall, this wide range of mineral profile in frass helps recycle nutrients by moving minerals from seafood waste into agricultural soils instead of losing them through disposal.

## Conclusion

This study demonstrates that mineral and trace element partitioning in BSF larvae and frass is dependent on the substrate. Calcium, manganese and zinc were accumulated by the larvae in varying extent. Elements such as aluminium, iron, boron, and copper did not accumulate in the larval tissues and concentrated in the frass. This pattern of nutrient flow is suggestive of the dual benefit of BSF bioconversion. The frass accumulates elemental minerals that can be slowly mobilized with soil pH shifts, while the larvae stay low in minerals and metals of concern. This not only ensures the safety of larvae as a feed ingredient but also confirms that seafood waste substrate can be used in BSF systems without adverse effects. Though these seafood waste substrates remained low in heavy metal concentrations, as seen in Table 1, any future bioconversion study of waste materials should have thorough analysis of heavy metals to ensure feed safety and reduce contamination risk in soils. In addition, it also provides insight into customizing diet blends based on elemental concentrations of waste streams to serve specific functions for feedstocks and fertilizers. As the use BSF larvae and frass increases, proactive decision can drive valorisation opportunities in local areas to increase micronutrient availability for livestock or plant agricultural systems. Future studies should test how readily these minerals in frass are available with controlled greenhouse trials for ornamental plants, evaluate crop and soil responses under field conditions for vegetables and fruits, and explore the use of additional seafood waste streams to assess the safety and consistency of mineral partitioning during BSF bioconversion.

## Author contributions

Shristi Upadhyaya: conceptualization, methodology, validation, formal analysis, investigation, data curation, writing – original draft, visualization. Michael Hayes: conceptualization, methodology, validation, investigation, writing – review & editing, visualization, supervision, project administration, funding acquisition. Victoria Vail: methodology, investigation, resources. Damian Tweedy: conceptualization, methodology, investigation, resources, writing – review & editing. Erik Neff: conceptualization, methodology, validation, investigation, resources, writing – review & editing, project administration. Jeffrey Plumlee: writing – review & editing, supervision, project administration, funding acquisition. Damon Abdi: writing – review & editing, supervision, project administration, funding acquisition.

## Conflicts of interest

At the time of this trial, authors VV, DT, and EN were employed by Fluker's Cricket Farm, Inc., an insect rearing company. The authors provided technical expertise and resources and did not have a role in the analysis of the data. The other authors declare that they have no known competing financial interests or personal relationships that could have appeared to influence the work reported in this paper.

## Supplementary Material

RA-016-D6RA06918E-s001

## Data Availability

Data will be made available on request. Supplementary information (SI) is available. See DOI: https://doi.org/10.1039/d6ra06918e.
