# Supplementary material for: Element partitioning of minerals and trace metals in value-added product from insect-mediated valorisation of seafood waste
Source: RSC Adv. 2026 Sep 16;16(44):55322–33. doi: 10.1039/d6ra06918e (PMC13579091; doi:10.1039/d6ra06918e)
Supplement: Supplementary file 1 [file RA-016-D6RA06918E-s001.pdf]

Supplementary Information (SI) for RSC Advances.
This journal is © The Royal Society of Chemistry 2026

Electronic Supplemental Information: Element partitioning of minerals and trace metals in value-added product from insect-mediated valorisation of seafood waste

*Shristi Upadhyaya[a], *Michael Hayes[a,b], Victoria Vail[c], Damian Tweedy[c], Erik Neff[c], Jeffrey Plumlee [b,d], and Damon Abdi[e]*

[a] *School of Plant, Environmental and Soil Sciences, Louisiana State University Agricultural Center, 219 Sturgis Hall, Baton Rouge, LA, 70803, USA*
[b] *Louisiana Sea Grant, Louisiana State University, Baton Rouge, LA, 70803, USA*
[c] *Fluker's Cricket Farm, Inc., 1333 Plantation Avenue, Port Allen, LA 70767, USA*
[d] *School of Renewable Natural Resources, Louisiana State University Agricultural Center, 334 Renewable Natural Resources Building, Baton Rouge, LA, 70803, USA*
[e] *Louisiana State University Agricultural Center Hammond Research Station, 21549 Old Covington Hwy, Hammond, LA, 70403, USA*

**Corresponding Author (mhayes@agcenter.lsu.edu)*

Page Number: 5
Figures: 0
Tables: 7

Supplemental Material

All analysis under this investigation were conducted by a certified service lab, Agricultural Chemistry Laboratory, at Louisiana State University. The service lab follows all standard laboratory methodologies and reports on their methods on the public facing website. Elemental profiling was performed using Inductively Coupled Plasma (ICP) spectroscopy under two distinct groupings. Group 1 elements are classified as essential macro- and micronutrients such as boron, calcium, copper, iron, magnesium, manganese, phosphorus, potassium, sodium, sulfur, and zinc. Group 2 elements comprised of aluminum, barium, cadmium, chromium, cobalt, lead, molybdenum, nickel, selenium, and arsenic. Both ICP group 1 and group 2 elements were analyzed with EPA method 200.7, EPA Method 3052, and AOAC Method 985.0, depending on the element.[1,2,3] The compounds excluded from discussion due to low levels and insignificant trends are listed in the supplementary tables below:

Table 1. *Elemental metal composition of shrimp-based feed formulations reported on dry matter basis.*

Parameters	100CC (Mean±SD)	25S75CC (Mean±SD)	40S60CC (Mean±SD)	50S50CC (Mean±SD)	60S40CC (Mean±SD)
**Molybdenum (PPM)**	1.98 ± 0.08	2.26 ± 0.09	2.45 ± 0.83	2.11 ± 0.10	2.18 ± 0.15
**Selenium (PPM)**	12.12 ± 0.03	12.07 ± 0.10	12.17 ± 0.02	11.98 ± 0.15	12.26 ± 0.50
**Cobalt (PPM)**	0.86 ± 0.10	1.18 ± 0.02	0.78 ± 0.04	0.75 ± 0.03	1.07 ± 0.11
**Arsenic (PPM)**	6.06 ± 0.01	6.09 ± 0.06	6.01 ± 0.11	5.99 ± 0.14	6.03 ± 0.22
**Nickel (PPM)**	2.11 ± 0.33	2.40 ± 0.82	2.32 ± 0.72	4.10 ± 3.42	1.68 ± 0.17
**Lead (PPM)**	5.04 ± 0.08	5.02 ± 0.05	5.13 ± 0.09	4.95 ± 0.14	5.05 ± 0.28
**Chromium (PPM)**	1.93 ± 0.06	2.14 ± 0.21	2.20 ± 0.19	2.03 ± 0.15	2.26 ± 0.29
**Cadmium (PPM)**	0.80 ± 0.01	0.79 ± 0.01	0.79 ± 0.01	0.81 ± 0.02	0.82 ± 0.02

Table 2. *Elemental metal composition of crab-based feed formulations reported on dry matter basis.*

Parameters	100CC (Mean±SD)	25S75CC (Mean±SD)	40S60CC (Mean±SD)	50S50CC (Mean±SD)	60S40CC (Mean±SD)
**Molybdenum (PPM)**	2.28 ± 0.06	2.47 ± 0.26	2.07 ± 0.10	2.17 ± 0.07	1.98 ± 0.14
**Selenium (PPM)**	13.28 ± 0.81	15.46 ± 1.52	16.54 ± 0.59	16.04 ± 0.62	16.23 ± 0.94
**Cobalt (PPM)**	0.66 ± 0.04	0.77 ± 0.08	0.83 ± 0.03	0.80 ± 0.03	0.81 ± 0.05
**Arsenic (PPM)**	6.64 ± 0.41	7.73 ± 0.76	8.27 ± 0.29	8.02 ± 0.31	8.11 ± 0.47
**Nickel (PPM)**	2.74 ± 0.17	2.14 ± 0.19	1.86 ± 0.17	1.63 ± 0.39	1.40 ± 0.12
**Lead (PPM)**	5.64 ± 0.35	6.57 ± 0.65	7.03 ± 0.25	6.82 ± 0.26	6.90 ± 0.40
**Chromium (PPM)**	2.90 ± 0.42	3.06 ± 0.38	2.97 ± 0.43	3.99 ± 1.52	3.56 ± 0.12
**Cadmium (PPM)**	1.00 ± 0.06	1.16 ± 0.11	1.24 ± 0.04	1.20 ± 0.05	1.22 ± 0.07

Table 3. *Elemental metal composition of larvae reared on shrimp-based diets reported on dry matter basis.*

Parameters	100CC (Mean±SD)	25S75CC (Mean±SD)	40S60CC (Mean±SD)	50S50CC (Mean±SD)	60S40CC (Mean±SD)
**Molybdenum (PPM)**	1.95 ± 0.04	1.87 ± 0.06	2.13 ± 0.07	2.01 ± 0.10	2.05 ± 0.06
**Selenium (PPM)**	8.56 ± 0.18	10.05 ± 0.42	10.86 ± 0.37	9.54 ± 0.52	9.31 ± 0.50
**Cobalt (PPM)**	0.43 ± 0.01	0.50 ± 0.02	0.54 ± 0.02	0.48 ± 0.03	0.48 ± 0.03
**Arsenic (PPM)**	4.28 ± 0.09	5.03 ± 0.21	5.43 ± 0.19	4.77 ± 0.26	4.66 ± 0.25
**Nickel (PPM)**	0.77 ± 0.04	0.75 ± 0.03	0.87 ± 0.06	0.75 ± 0.07	1.19 ± 0.07
**Lead (PPM)**	3.64 ± 0.08	4.27 ± 0.18	4.62 ± 0.16	3.98 ± 0.24	3.96 ± 0.21
**Chromium (PPM)**	1.28 ± 0.10	1.11 ± 0.06	1.26 ± 0.02	1.27 ± 0.04	1.51 ± 0.08
**Cadmium (PPM)**	0.64 ± 0.01	0.75 ± 0.03	0.81 ± 0.03	0.72 ± 0.04	0.70 ± 0.04

Table 4. *Elemental metal composition of larvae reared on crab-based diets reported on dry matter basis.*

Parameters	100CC (Mean±SD)	25S75CC (Mean±SD)	40S60CC (Mean±SD)	50S50CC (Mean±SD)	60S40CC (Mean±SD)
**Molybdenum (PPM)**	1.86 ± 0.04	2.15 ± 0.04	1.58 ± 0.02	1.53 ± 0.04	1.51 ± 0.06
**Selenium (PPM)**	12.00 ± 0.01	12.60 ± 0.39	12.00 ± 0.01	11.99 ± 0.02	11.96 ± 0.02
**Cobalt (PPM)**	0.60 ± 0.0003	0.60 ± 0.0003	0.60 ± 0.0005	0.60 ± 0.0009	0.60 ± 0.001
**Arsenic (PPM)**	5.59 ± 0.004	5.65 ± 0.06	5.62 ± 0.02	5.60 ± 0.01	5.58 ± 0.01
**Nickel (PPM)**	1.50 ± 0.04	0.89 ± 0.08	1.35 ± 0.03	1.22 ± 0.05	1.21 ± 0.06
**Lead (PPM)**	5.00 ± 0.003	4.99 ± 0.003	5.00 ± 0.004	5.00 ± 0.01	4.99 ± 0.01
**Chromium (PPM)**	1.61 ± 0.17	1.42 ± 0.07	1.50 ± 0.04	1.35 ± 0.02	1.58 ± 0.05
**Cadmium (PPM)**	0.80 ± 0.0005	0.80 ± 0.0006	0.80 ± 0.0004	0.80 ± 0.001	0.80 ± 0.001

Table 5. *Elemental metal composition of frass from shrimp-based diets reported on dry matter basis.*

Parameters	100CC (Mean±SD)	25S75CC (Mean±SD)	40S60CC (Mean±SD)	50S50CC (Mean±SD)	60S40CC (Mean±SD)
**Molybdenum (PPM)**	4.34 ± 0.07	4.51 ± 0.23	4.64 ± 0.18	3.91 ± 0.40	3.73 ± 0.02
**Selenium (PPM)**	8.40 ± 0.80	6.85 ± 0.51	8.65 ± 0.39	7.08 ± 1.26	8.53 ± 0.80
**Cobalt (PPM)**	2.29 ± 0.16	3.25 ± 0.14	3.17 ± 0.16	1.71 ± 0.11	1.64 ± 0.07
**Arsenic (PPM)**	4.20 ± 0.40	3.42 ± 0.25	4.32 ± 0.20	3.54 ± 0.63	4.27 ± 0.40
**Nickel (PPM)**	4.52 ± 0.33	6.52 ± 1.10	7.92 ± 1.38	6.36 ± 0.99	8.35 ± 0.73
**Lead (PPM)**	3.57 ± 0.34	2.91 ± 0.22	3.68 ± 0.17	3.01 ± 0.54	3.63 ± 0.34
**Chromium (PPM)**	5.03 ± 0.48	9.96 ± 2.50	13.56 ± 3.08	10.27 ± 1.77	16.91 ± 2.38
**Cadmium (PPM)**	0.63 ± 0.06	0.51 ± 0.04	0.65 ± 0.03	0.53 ± 0.09	0.64 ± 0.06

Table 6. *Elemental metal composition of frass from crab-based diets reported on dry matter basis.*

Parameters	100CC (Mean±SD)	25S75CC (Mean±SD)	40S60CC (Mean±SD)	50S50CC (Mean±SD)	60S40CC (Mean±SD)
**Molybdenum (PPM)**	4.22 ± 0.06	4.03 ± 0.16	3.26 ± 0.02	2.87 ± 0.09	2.57 ± 0.05
**Selenium (PPM)**	11.98 ± 0.02	11.99 ± 0.01	11.97 ± 0.01	12.05 ± 0.08	12.08 ± 0.10
**Cobalt (PPM)**	2.12 ± 0.05	1.92 ± 0.09	1.30 ± 0.02	1.10 ± 0.01	0.79 ± 0.01
**Arsenic (PPM)**	5.59 ± 0.01	5.60 ± 0.00	5.59 ± 0.01	5.73 ± 0.14	5.92 ± 0.13
**Nickel (PPM)**	6.30 ± 0.29	5.02 ± 0.27	3.42 ± 0.09	2.83 ± 0.07	2.09 ± 0.01
**Lead (PPM)**	4.99 ± 0.01	5.00 ± 0.01	4.99 ± 0.003	4.98 ± 0.005	4.99 ± 0.01
**Chromium (PPM)**	7.92 ± 0.64	6.45 ± 0.51	5.20 ± 0.03	4.72 ± 0.14	4.36 ± 0.14
**Cadmium (PPM)**	0.80 ± 0.002	0.80 ± 0.002	0.80 ± 0.0003	0.80 ± 0.0004	0.80 ± 0.0008

Table 7. *Separate two-way fixed-effects ANOVAs for elemental concentrations in larvae and frass*

Matrix	Element	Substrate		Inclusion level		Substrate × inclusion	
		F (1, 16)	p	F (3, 16)	p	F (3, 16)	p
**Larvae**	B	0.11	0.743	3.33	0.046	1.71	0.205
**Larvae**	Ca	147.30	<0.001	3.99	0.027	0.65	0.593
**Larvae**	Cu	1.85	0.193	11.75	<0.001	6.15	0.006
**Larvae**	Fe	23.49	<0.001	4.48	0.018	3.23	0.0502
**Larvae**	Mn	23.54	<0.001	132.68	<0.001	2.37	0.108
**Larvae**	Zn	74.26	<0.001	20.14	<0.001	11.15	<0.001
**Larvae**	Al	94.39	<0.001	40.78	<0.001	2.00	0.154
**Larvae**	Ba	290.54	<0.001	3.82	0.031	2.02	0.152
**Frass**	B	8.45	0.010	11.07	<0.001	1.58	0.233
**Frass**	Ca	449.26	<0.001	198.79	<0.001	30.78	<0.001
**Frass**	Cu	84.20	<0.001	77.43	<0.001	15.98	<0.001
**Frass**	Fe	250.29	<0.001	53.48	<0.001	26.92	<0.001
**Frass**	Mn	608.66	<0.001	43.05	<0.001	26.41	<0.001
**Frass**	Zn	17.69	<0.001	111.80	<0.001	11.15	<0.001
**Frass**	Al	10.70	0.005	2.06	0.146	1.84	0.181
**Frass**	Ba	1119.77	<0.001	73.56	<0.001	28.62	<0.001

References:

1. U.S. Environmental Protection Agency., 1994. *Method 200.7: Determination of Metals and Trace Elements in Water and Wastes by Inductively Coupled Plasma-Atomic Emission Spectrometry*. https://www.epa.gov/esam/method-2007-determination-metals-and-trace-elements-water-and-wastes-inductively-coupled

2. U.S. Environmental Protection Agency., 1996. *Method 3052: Microwave Assisted Acid Digestion of Siliceous and Organically Based Matrices*. https://www.epa.gov/sites/default/files/2015-12/documents/3052.pdf

3. AOAC INTERNATIONAL., 2023b. AOAC Official Method 985.01: Metals and Other Elements in Plants and Pet Foods: Inductively Coupled Plasma (ICP) Spectroscopic Method. In G. W. Latimer, Jr. (Ed.), *Official Methods of Analysis of AOAC INTERNATIONAL* (22nd ed.). AOAC Publications. https://doi.org/10.1093/9780197610145.003.1316
